# An extended car-following model at un-signalized intersections under V2V communication environment

**DOI:** 10.1371/journal.pone.0192787

**Published:** 2018-02-09

**Authors:** Tao Wang, Jing Zhao, Peng Li

**Affiliations:** 1 China Institute of Urban Governance, Shanghai Jiao Tong University, Shanghai, P.R.China; 2 Department of Traffic Engineering, University of Shanghai for Science and Technology, Shanghai, P.R.China; 3 Department of Supply Chain Management, Rutgers University, The State University of New Jersey, Newark, New Jersey, United States of America; Chongqing University, CHINA

## Abstract

An extended car-following model is proposed in this paper to analyze the impacts of V2V (vehicle to vehicle) communication on the micro driving behavior at the un-signalized intersection. A four-leg un-signalized intersection with twelve streams (left-turn, through movement, and right turn from each leg) is used. The effect of the guidance strategy on the reduction of the rate of stops and total delay is explored by comparing the proposed model and the traditional FVD car-following model. The numerical results illustrate that potential conflicts between vehicles can be predicted and some stops can be avoided by decelerating in advance. The driving comfort and traffic efficiency can be improved accordingly. More benefits could be obtained under the long communication range, low to medium traffic density, and simple traffic pattern conditions.

## Introduction

### Background

Traffic congestion at intersections continues to worse in many cities. With the development of the intelligent traffic system, the V2V (vehicle to vehicle) communication technology has become an alternative method to improve the running efficiency of the road. By providing drivers the actual running status of surrounding vehicles, the drivers can adjust their driving speed in advance to avoid the sudden stop or acceleration. Therefore, the micro driving behavior may be affected by the V2V communication.

In the research field of micro driving behavior, abundant models with traffic phenomena and driving behavior have been explored by many authors [1–3], which includes two categories: microscopic traffic flow models [4–19] and macroscopic models [20–38]. The former simulates single vehicle-driver units and represents vehicular microscopic properties, such as position, velocity, acceleration, and deceleration; while the later formulates the relationships among traffic flow characteristics, such as density, traffic flow, speed. Researchers use theses traffic flow models to simulate the complex traffic condition under different traffic pattern, geometric, control, and management conditions.

The study builds on earlier work on car-following model with consideration of vehicle to vehicle communication by Tang [39] and Zhao [40]. In this model, we extend the work of Zhao [40] that only focus on two conflicting streams to an un-signalized intersection with twelve streams (left-turn, through movement, and right turn from each leg).

### Related literature

The large literature on traffic flow model that consider the special driving environment of V2V communication to analyze the micro driving behavior is explored by many authors [39–46]. Ngoduy et al. [41] modeled the dynamics of cooperative traffic flow by continuum approach. The adjustment of the speed of the equipped vehicle to the speed of downstream congested traffic is suggested. The influence of the penetration rate of the equipped vehicles on traffic flow stability and capacity in a freeway is analyzed. Knorr et al. [42] also presented a strategy on determining how and when to change driving behavior under theV2V communication environment. Both above two studies show that operational efficiency can be increased even under a low ratio of equipped vehicles. Mahmassani [43] further analyzed the impacts of autonomous vehicles and connected vehicle system on traffic operations. A microsimulation framework is introduced to feature the varying behavioral mechanisms of different vehicles, which can be used to analyze how connected vehicles improve the throughput and stability of a traffic facility. Liu [44] proposed a data analytic methodology to extract critical information from raw basic safety messages data. The critical information includes the driver’s own driving behaviors and the potential dangers in surrounding roadways. Sun [45, 46] and Kamal [47] proposed some significant vehicle velocity models for predictive energy management and then extended the model as investigating adaptive-ECMS with velocity forecast ability for Hybrid Electric Vehicles. However, how the V2V communication environment affects the micro driving behavior were not considered and analyzed. Jia [48] established a novel platoon-based cooperative driving model under the V2V communication environment. Both theoretical analysis and simulation results showed that the globally achievable leader’s information plays a critical role in stabilizing the platoon-based cooperative driving system. Tang [39] established a new car following model to investigate the micro driving behavior of the vehicles that can communicate with each other. For daily traffic operation, Zhao [40] extended an car-following model under V2V communication environment to explore the driving behavior for two crossing streams.

In the urban street system, intersections are the most complex individual locations [49–52], which normally contains twelve movements. Although much is known on the positive effect of the V2V communication on the macroscopic traffic flow, the microscopic driving behavior has not been deeply discussed, especially at intersections. In this paper, the V2V communication technology is used to aware drivers the potential conflicts at the oncoming un-signalized intersection. Hence, it is possible for vehicles to adjust speed in advance, and then go through the intersection more smoothly. A car-following model is established to explore the adjustment of driving behavior.

### Research motivation

In light of the above, this study aims to develop a novel model to extend the exist car-following model by Tang [39] and Zhao [40], in which all the twelve streams (left-turn, through movement, and right turn from each leg) at an un-signalized intersection are considered. Based on the proposed model, the V2V sensitively analysis was conducted. Form numerical results, it is observed that the potential conflict between vehicles can be predicated, then some stop can be avoided by decelerating in advance. Moreover, the proposed model can guide the drivers to adjust their driving behavior to obtain more comfortable driving environment and improve traffic operational efficiency. The main effects on stop rate and total delay time with V2V communication environment at un-signalized intersection, which are communication rang, the average initial space headway, and the through movement percentage.

## Proposed model

**Fig 1:**
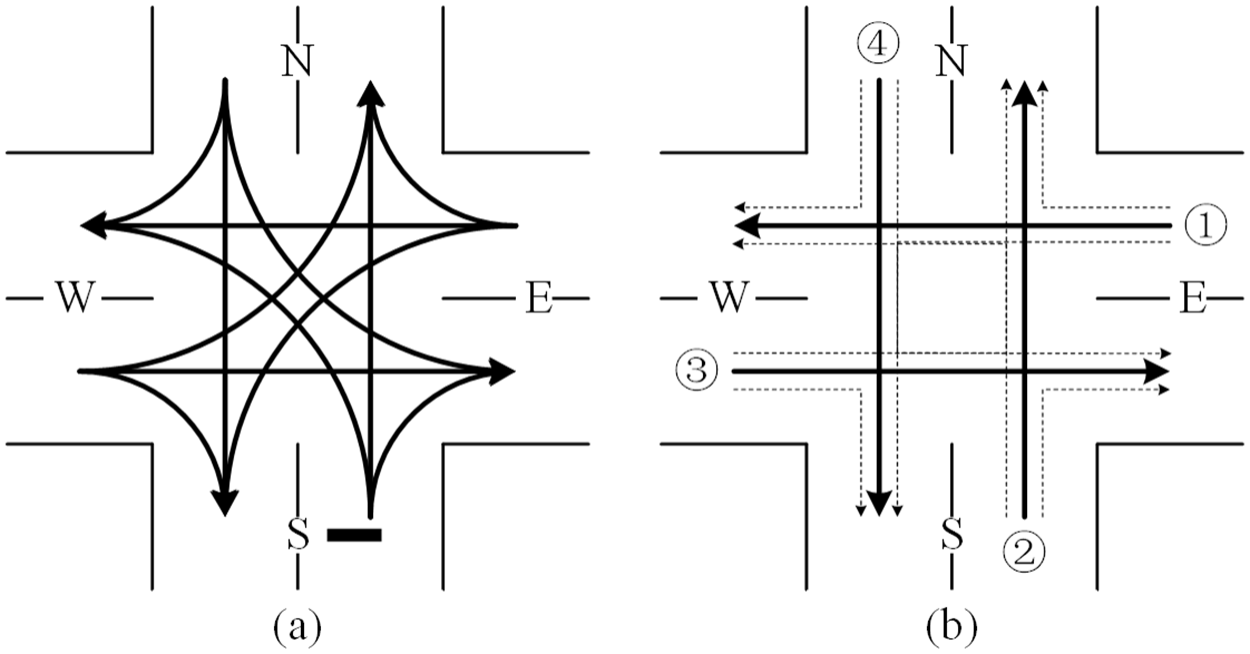
Target un-signalized intersection. (a) Original (b) Simplified.

A four-leg un-signalized intersection with twelve movements (three movements in each approach) is used, as illustrated in Fig 1(a). For easy modeling, it can be simplified as Fig 1(b). The four through movements are numbered, as shown the solid arrow in Fig 1(b). The turning movements can be denoted as the combination of two through movements (e.g., the left turn from east approach can be denoted as movement 1 + movement 4).

The existing car-following models on a single lane can be described as follows:

$$\frac{\text{d}v_{n}(t)}{\text{d}t}=f\left(v_{n}(t),\Delta x_{n}(t),\Delta v_{n}(t),\ldots \right),$$ (1)

where *f*(•) is the stimulus function of vehicle *n* at time *t*; *v*_*n*_(*t*) is the speed of vehicle *n* at time *t*, m/s; Δ*x*_*n*_(*t*) = *x*_*n*−1_(*t*) − *x*_*n*_(*t*) is the space distance between vehicle *n* and vehicle *n*-1 at time *t*, m; Δ*v*_*n*_(*t*) = *v*_*n*−1_(*t*) − *v*_*n*_(*t*) is the speed difference between vehicle *n* and vehicle *n*-1 at time *t*, m/s;.

The *f*(•) can be determined by many factors, such as the speed, headway, and relative speed. With different expression, the existing car-following models can be divided into three categories, namely (1) optimal velocity (OV) model [53], (2) full velocity difference (FVD) model [54], and (3) full velocity and acceleration difference (FVAD) model [13]. In this paper, the new car-following model is established based on the traditional FVD model, as shown in Eq (2).

$$\frac{\text{d}v_{n}(t)}{\text{d}t}=\kappa \left(V\left(\Delta x_{n}(t)\right)-v_{n}(t)\right)+\lambda \Delta v_{n}(t),$$ (2)

where *V*(.) is the optimal speed function of vehicle *n*, m/s, which can be further determined by Eq (3) [55]; *κ* and *λ* are parameters.

$$V\left(\Delta x_{n}(t)\right)=V_{1}+V_{2}\text{tanh}\left(C_{1}\left(\Delta x_{n}(t)-l_{c}\right)-C_{2}\right),$$ (3)

where *l*_*c*_ is the length of the vehicle, m; *V*_1_, *V*_2_, *C*_1_, *C*_2_ are parameters.

**Fig 2:**
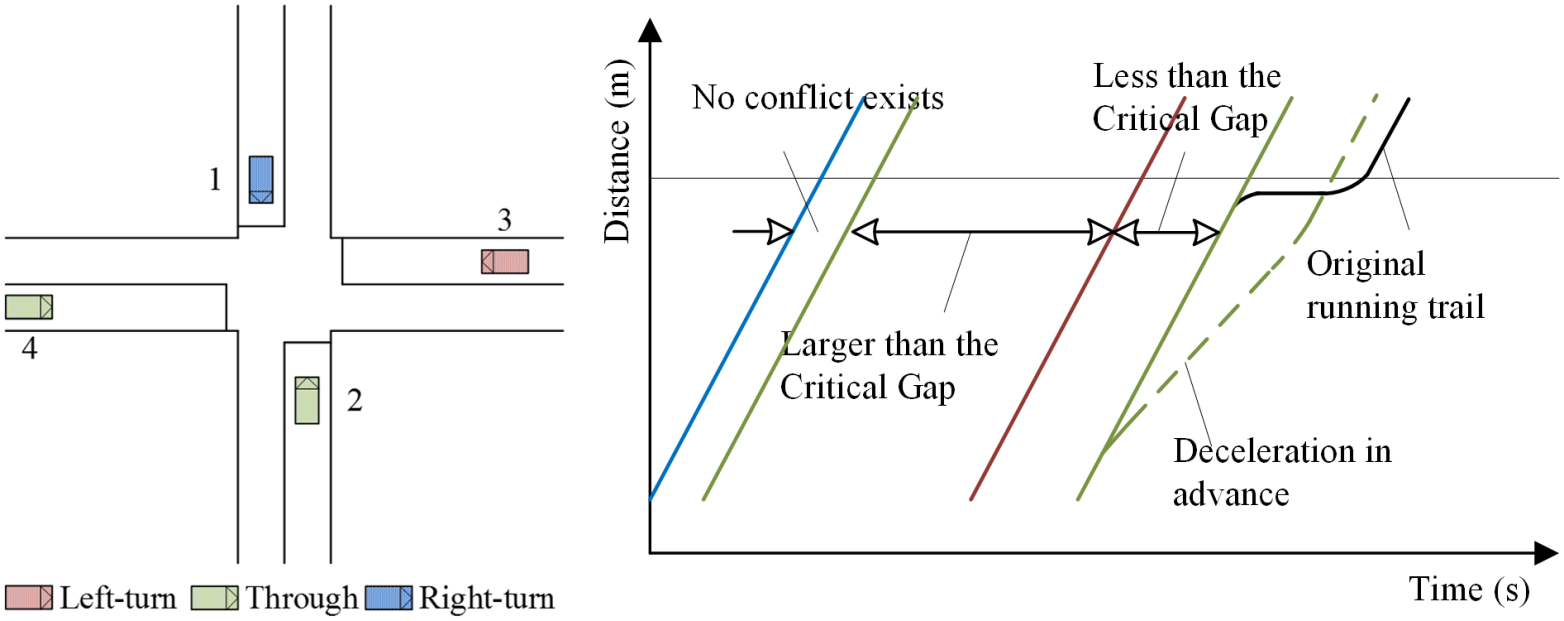
Schematic diagram of identifying and avoiding conflicts.

Different from the traditional operation environment, the V2V communication technology provides the information of the speed and position of all the equipped vehicles within the communication range. Therefore, the sequence of vehicles to go through the intersection can be decided and the potential conflicts can be predicted in advance. Instead of stopping and waiting for consensus with the conflicting vehicle as the all-way stop controlled intersections, vehicles can decelerate in advance and avoid the conflicts, as illustrated in Fig 2. Whether there is a conflict with each pair of vehicles can be judged by comparing the gap between each pair of vehicles and the critical gap at the intersection (gray line). E.g., for the vehicle 1 and 2, there is no conflict exits because the two traffic flows are not conflicting. For the vehicle 2 and 3, there is no conflict exits because the gap between vehicle 2 and 3 is larger than the critical gap. For the vehicle 3 and 4, there is a conflict because the gap between vehicle 3 and 4 is less than the critical gap.

The assumptions of the proposed models include:

1. Both two crossing streets of the intersection are two-lane street with one lane for each direction.
2. The priority rank of all the traffic streams in the intersection is equal.
3. The vehicles that come first should be serviced first.
4. The overtaking is forbidden.
5. The speed and position of all the vehicles within the communication range are known.

The algorithm for identifying and avoiding conflicts includes the following four steps:

Step 1: identifying the potential conflicts. For any pair of vehicles, Eq (4) can be used to identify whether the two traffic streams are conflicting in space.

$$\alpha_{mn}=\{\begin{array}{ll} 1 & d_{n}=d_{m}\\ 1 & o_{n}=d_{n}=o_{m}+1\\ 1 & o_{n}=d_{n}=d_{m}-1\\ 1 & o_{n}=d_{n}+1=o_{m}+2=d_{m}+2\\ 1 & o_{n}=d_{n}+1=o_{m}+1=d_{m}+2\\ 1 & o_{n}=d_{n}+1=o_{m}-1=d_{m}\\ 1 & o_{n}=d_{n}+1=o_{m}-1=d_{m}-1\\ 0 & \text{others} \end{array},$$ (4)

where *α*_*mn*_ is a binary variable showing whether the pair of vehicles (m, n) is conflicting, 1-Yes, 0-No; *o*_*n*_ is the origin stream of vehicle *n*, as shown in Fig 1; *d*_*n*_ is the destination stream of vehicle *n*, as shown in Fig 1.

Step 2: judging whether the time gap between vehicle *n* and the nearest front conflicting vehicle satisfies the critical safety gap, as shown in Eq (5). If yes, turn to Step 3; otherwise, turn to Step 4.

$$h_{n}(t)=\text{min}\left\{\frac{x_{m}(t)}{v_{m}(t)}-\frac{x_{n}(t)}{v_{n}(t)}\right\}\geq c,\frac{x_{m}(t)}{v_{m}(t)}-\frac{x_{n}(t)}{v_{n}(t)}\geq 0,$$ (5)

where *h*_*n*_(*t*) is the minimum headway between vehicle *n* and the front conflicting vehicles, s; *c* is the critical safety gap, s.

Step 3: no change of driving behavior should be made. The car-following model could be given by Eq (6).

$$a_{n1}(t)=\kappa \left(V\left(\Delta x_{n}(t)\right)-v_{n}(t)\right)+\lambda \Delta v_{n}(t),h_{n}(t)\geq c,x_{I}-x_{n}(t)\leq D,$$ (6)

where *a*_*n*1_(*t*) is the traditional FVD model; *x*_*I*_ is the position of the intersection, m; *D* is the V2V communication range, m.

Step 4: the nth vehicle should decelerate and follow the conflicting vehicle, as shown in Eq (7). For smooth running purpose, the deceleration and the minimum velocity should be limited, as shown in Eqs (8) and (9), respectively.

$$\begin{array}{c} a_{n2}(t)=\kappa \left(V\left(\Delta x_{n,m}(t)\right)-v_{n}(t)\right)+\lambda \Delta v_{n,m}(t) \end{array},h_{n}(t)<c,x_{I}-x_{n}(t)\leq D,$$ (7)

where *a*_*n*2_(*t*) is the deceleration strategy model; Δ*x*_*n*,*m*_(*t*) = *x*_*m*_(*t*) − *x*_*n*_(*t*) is the relative space gap between vehicle *n* and the front conflicting vehicle *m* at time *t*, m; Δ*v*_*n*,*m*_(*t*) = *v*_*m*_(*t*) − *v*_*n*_(*t*) is the speed difference between vehicle *m* and vehicle *n* at time *t*, m/s.

$$\frac{\text{d}v_{n}(t)}{\text{d}t}\geq a_{\text{min}},h_{n}(t)\geq c,x_{I}-x_{n}(t)\leq D,$$ (8)

$$\frac{\text{d}v_{n}(t)}{\text{d}t}=0,h_{n}(t)\geq c,x_{I}-x_{n}(t)\leq D,v_{n}(t)\leq v_{\text{min}},$$ (9)

where *a*_min_ is the minimum deceleration, m/s^2^; *v*_min_ is the minimum velocity, m/s.

In summary, the extended car-following model at the un-signalized intersection under the V2V communication environment can be described as follows:

$$\left\{ \begin{array}{l} \frac{\text{d}v_{n}(t)}{\text{d}t}=a_{n1}(t),x_{I}-x_{n}(t)>D_{}\;or_{}\;x_{n}(t)>x_{I}\\ \frac{\text{d}v_{n}(t)}{\text{d}t}=a_{n1}(t),x_{I}-x_{n}(t)\leq D,h_{n}(t)\geq c\\ \begin{array}{c} \frac{\text{d}v_{n}(t)}{\text{d}t}=\text{min}\left\{\text{min}\left(a_{n2}(t),a_{\text{min}}\right),a_{n1}(t)\right\} \end{array}x_{I}-x_{n}(t)\leq D,h_{n}(t)<c\\ \frac{\text{d}v_{n}(t)}{\text{d}t}=\text{min}\left\{0,a_{n1}(t)\right\},x_{I}-x_{n}(t)\leq D,h_{n}(t)<c,v_{n}(t)=v_{\text{min}} \end{array}\right.$$ (10)

## Numerical tests

The improvement of the operational performance gained from the V2V communication techniques at un-signalized intersections are analyzed based on the proposed model in this section. The analysis contains the following two parts. First, the vehicles’ running trail under the proposed model are analyzed and compared with the traditional FVD model to show the advantage of the speed guidance strategy. Second, the sensitivity analysis was conducted to analyze the changing tendency of the performance with some key factors, including the communication range, traffic flow density, and traffic pattern.

Extensive numerical tests are used to analyze the proposed model. Since it is difficult to obtain the analytical solution of Eq (10), the proposed model is accomplished and simulated in Matlab. The speed and position of each vehicle can be calculated by the Euler forward difference, as shown in Eqs (11) and (12), respectively.

$$v_{\text{n}}\left(t+\Delta t\right)=v_{\text{n}}\left(t\right)+\Delta t\frac{\text{d}v_{\text{n}}\left(t\right)}{\text{d}t}$$ (11)

$$x_{\text{n}}\left(t+\Delta t\right)=x_{\text{n}}\left(t\right)+v_{\text{n}}\left(t\right)\Delta t+\frac{1}{2}\frac{\text{d}v_{\text{n}}\left(t\right)}{\text{d}t}{\left(\Delta t\right)}^{2}$$ (12)

where Δ*t* is the time-step length, s.

### Performance of the proposed model

A numerical simulation with 24 vehicles at an un-signalized intersection was used to analyzed the performance of the proposed model. The initial location, origin stream, destination stream, and moving direction of vehicles are listed in Table 1. The values of the parameters used in the test are set as follows: *x*_*I*_ = 1500 m, Δ*t* = 0.1 s, *κ* = 0.41 s^-1^, *λ* = 0.2 s^-1^, *l*_*c*_ = 9 m, *V*_1_ = 6.75 m/s, *V*_2_ = 7.91 m/s, *C*_1_ = 0.13 m^-1^, *C*_2_ = 1.57 m^-1^, *c* = 3 s, *v*_max_ = 16.67 m/s, *v*_min_ = 6 m/s, *D* = 300 m.

**Table 1:** Initial position and moving direction of vehicles.

No.	Origin stream, *o*_*n*_	Destination stream, *d*_*n*_	Movement	Position (m)	No.	Origin stream, *o*_*n*_	Destination stream, *d*_*n*_	Movement	Position (m)
**1**	1	1	WB-TH	0	**13**	1	1	WB-TH	-800
**2**	2	1	NB-LT	-100	**14**	2	1	NB-LT	-810
**3**	3	2	EB-LT	-200	**15**	3	2	EB-LT	-820
**4**	4	4	SB-TH	-300	**16**	4	4	SB-TH	-830
**5**	1	1	WB-TH	-400	**17**	1	1	WB-TH	-840
**6**	3	3	EB-TH	-410	**18**	2	1	NB-LT	-850
**7**	2	2	NB-TH	-500	**19**	3	2	EB-LT	-860
**8**	4	4	SB-TH	-510	**20**	4	4	SB-TH	-870
**9**	1	4	WB-LT	-600	**21**	1	1	WB-TH	-950
**10**	3	2	EB-LT	-610	**22**	1	1	WB-TH	-1030
**11**	2	1	NB-LT	-700	**23**	1	1	WB-TH	-1100
**12**	4	3	SB-LT	-710	**24**	1	1	WB-TH	-1200

Note: the abbreviations EB, WB, NB, and SB refer to eastbound, westbound, northbound, and southbound, respectively; abbreviations LT, TH indicate the left-turn and through movement, respectively.

**Fig 3:**
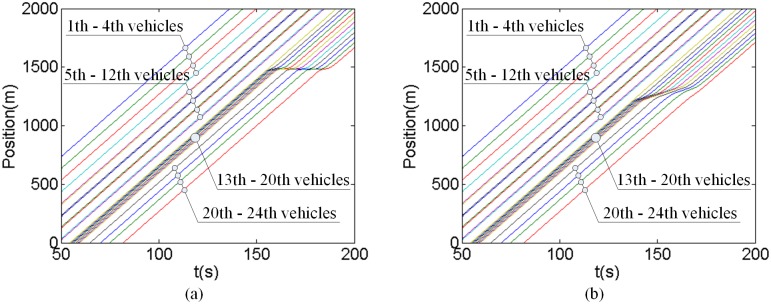
Each vehicle’s running trail. (a) FVD model (b) Proposed model.

**Fig 4:**
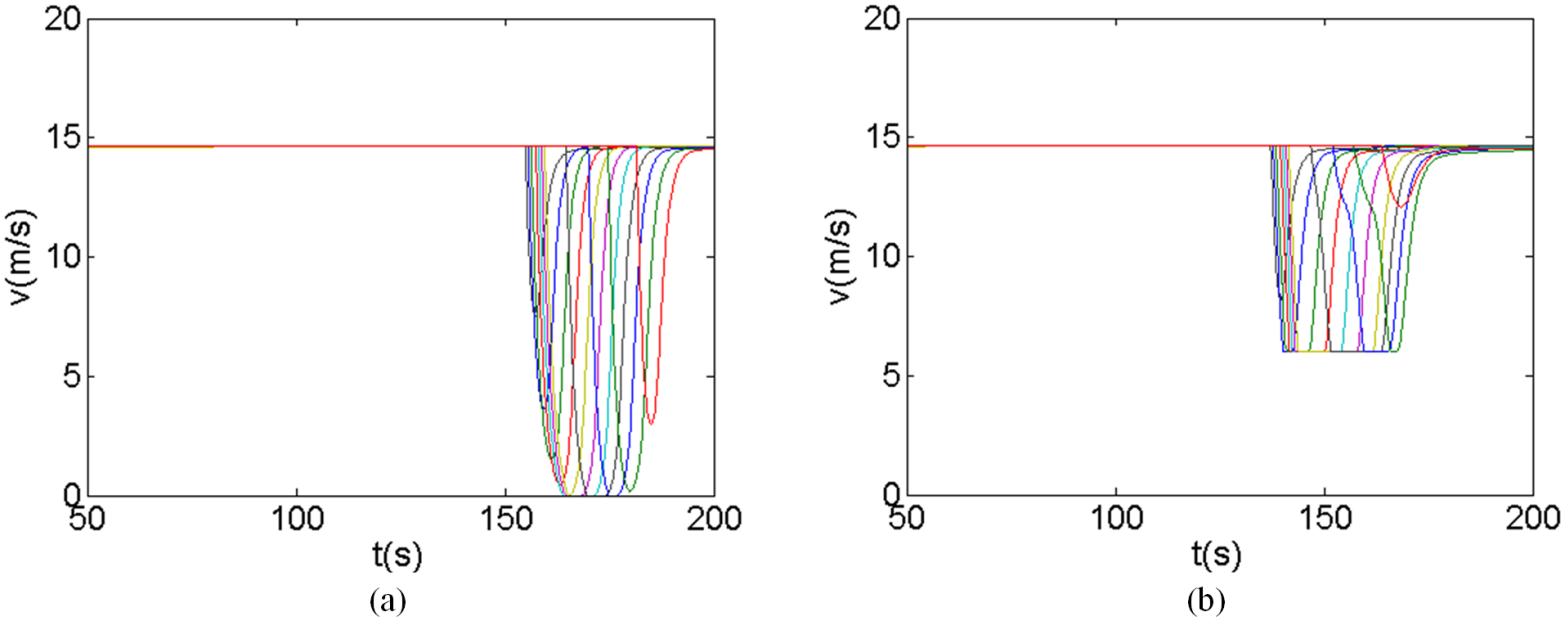
Each vehicle’s velocity. (a) FVD model (b) Proposed model.

Using the proposed model, the position and speed of each vehicle can be calculated, as illustrated in Figs 3 and 4, respectively. Comparing with the traditional FVD model, the following finding can be drawn:

1. For the first 4 vehicles, there are conflicts with each other. However, since the headways between them are larger than the critical safety gap, they can go through the intersection without decelerating. Their running trails are the same for the proposed model and tradition FVD model.
2. For the 5th and 6th vehicles, there are not conflicts with each other. Therefore, they can go through the intersection simultaneously. Along the same line, the pairs 7th and 8th vehicles, 9th and 10th vehicles, and 11th and 12th vehicles, go through the intersection simultaneously.
3. For the 13th to 20th vehicles, there are conflicts with each other and the headways between them are smaller than the critical safety gap. Therefore, under the tradition FVD model condition, they will stop at the stop line and wait until all the conflicting vehicles that touch the stop line earlier clear. However, under the proposed model condition, the potential conflict between these vehicles are predicted. They decelerate in advance and successfully pass the intersection without stopping, which can improve the driving comfort and traffic efficiency, and reduce the emissions.
4. For the 21th to 24th vehicles, there are not conflicts with each other. However, affected by the vehicle ahead, all these vehicles have to stop at the stop-line under the traditional FVD model. Under the V2V communication environment, the improved running efficiency of the 13th to 20th vehicles further bring benefits to the 21th to 24th vehicles. They can also go through the intersection more smoothly. The last (24th) vehicle even can go through the intersection without deceleration. Therefore, the V2V communication also is benefit not only to the conflicting vehicles but also to the following vehicles.

### Sensitivity analysis

The performance improvement gained from the V2V communication will be changed under different traffic and control conditions. The changing tendency of some key factors is discussed in this section. Two performance indices, including the rate of stopped vehicles and the total delay, were used to evaluate the performance. The average initial space headway is 50m. The percentage of the number of vehicles on each leg is equal (0.25 for each leg). The percentage of the number of vehicles for each movement is also equal (0.333 for each movement). The Other parameters were kept the same. For fair comparison, the initial position and turning movement of each vehicle are random. Under each testing scenario, 100 vehicles were used. Moreover, the model is simulated for 30 times under each testing scenario; and then the average value is used.

#### (1) Communication range

**Fig 5:**
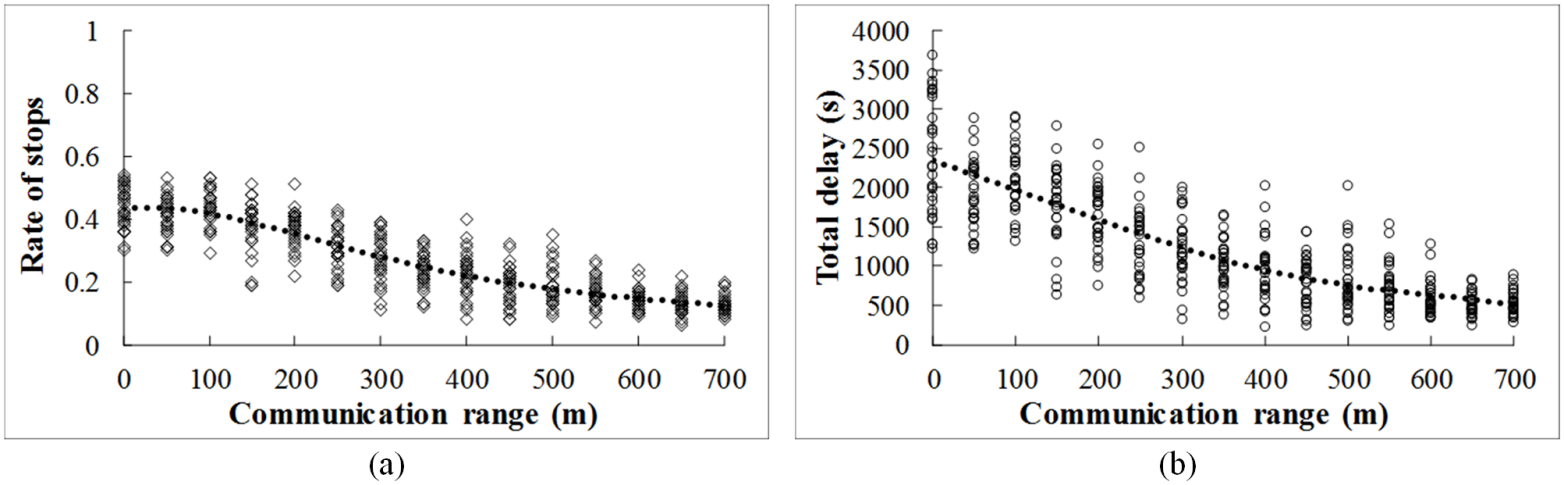
Effects of communication range. (a) Rate of stops (b) Total delay.

The communication range is changed from 0 m to 700m. The changing tendency of the rate of stops and total delay with the communication range is shown in Fig 5. Overall, more stops can be avoided when the communication range increases. It is due to the fact that the potential conflict can be identified earlier and there is longer distance for vehicle to decelerate and avoid the conflict. Hence, the total delay decreases accordingly. One can observe that the benefits (reduction in the rate of stops and total delay) caused by the V2V communication is sight when the communication range is less than 100m. The benefits become significant when the communication range is more than 100m. On average, 3.0% decrease in the rate of stops can be obtained by 50 m increase of the communication range. Compared with the no V2V communication condition (the communication range = 0), 50% and 80% stops can be avoided if the communication range is over 400m and 700m, respectively.

#### (2) Traffic flow density

**Fig 6:**
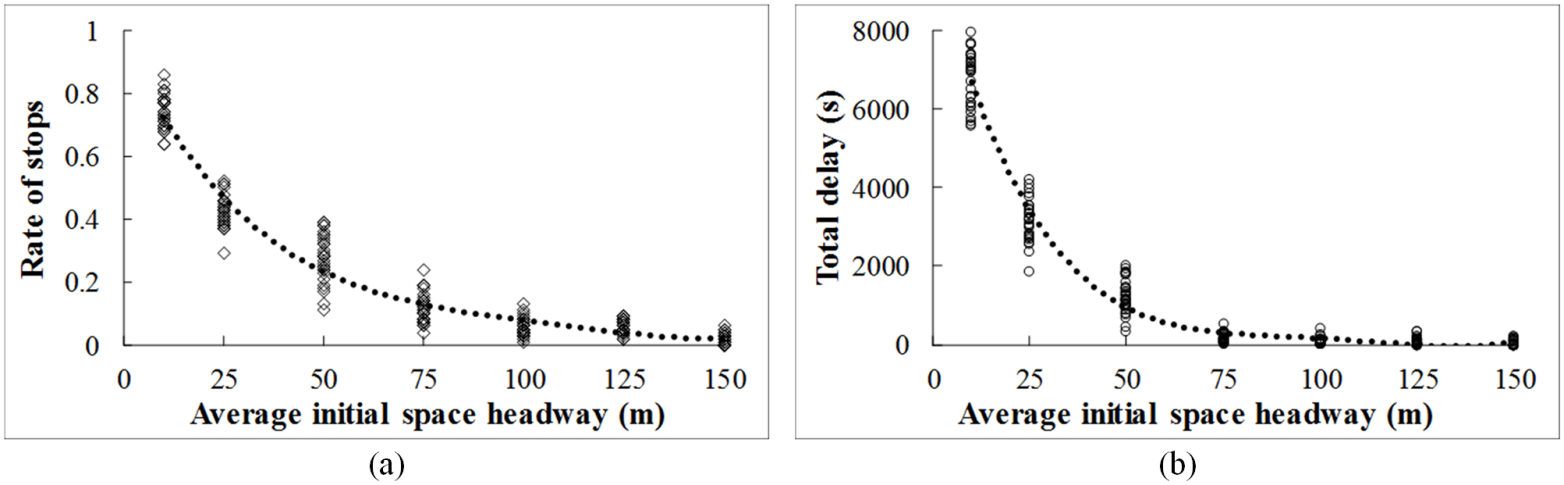
Effects of traffic flow density. (a) Rate of stops (b) Total delay.

The average initial space headway of the traffic flow is changed from 10 m to 150 m. The changing tendency of the rate of stops and total delay with the traffic flow density is shown in Fig 6. Overall, lower rate of stops and total delay can be obtained with the increase of the initial space headway (the decrease of the traffic flow density). It is due to the fact that there is no space redundancy to intervein vehicles of different movements by decelerating in advance when the traffic destiny is high. With the decrease of the traffic flow density, more space can be used for coordinating conflicting vehicles. Most of the stops (90%) can be avoided under the V2V communication environment when the average space headway of vehicles is larger than 75m.

#### (3) Traffic pattern

**Fig 7:**
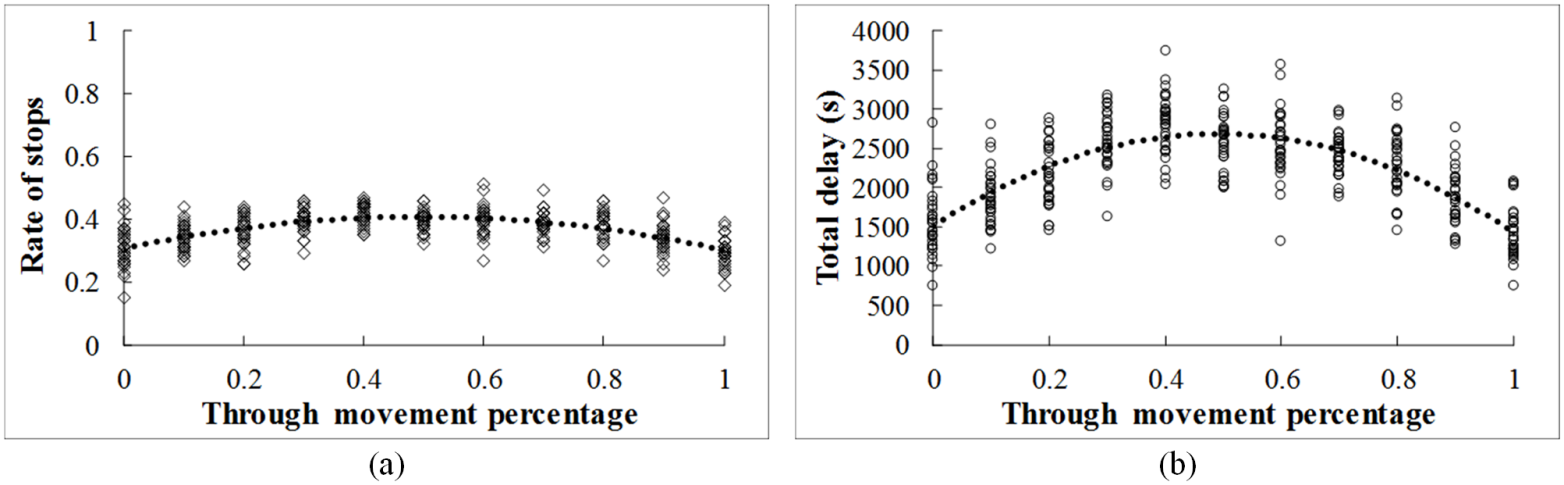
Effects of traffic pattern. (a) Rate of stops (b) Total delay.

One may wonder whether the changes in the volume percentage of different movements may affect the performance of the V2V communication. The through movement percentage is changed from 0% to 100%. The changing tendency of operational performance is shown in Fig 7. Overall, the rate of stops and total delay first increase and then decrease with the increase of the through movement percentage. The highest value occurs when the percentages of through and left turn are equal (50% to 50%). It is due to the fact that when the through movement percentage is quite low or high, the main traffic direction is a significant. Therefore, it will be easier to coordinate the traffic flow from different legs.

## Conclusions

In this paper, we propose an extended car-following model with consideration of V2V communication at un-signalized intersection with twelve streams (left-turn, through movement, and right turn from each leg) to analyze how the driving behavior and operational efficiency will be affected by different scenarios, such as communication rang between intelligent vehicles, average initial space headway of the traffic flow, and the volume percentage of different movement.

The numerical results show that the proposed model can qualitatively describe the effect of the V2V communication on microscopic driving behavior at un-signalized intersection. The potential conflict between vehicles can be predicted and some stops can be avoided by decelerating in advance. Therefore, the driving comfort and traffic efficiency can be improved.

The performance improvement gained from the V2V communication will be changed under different traffic and control conditions. Lower rate of stops and total delay can be obtained with the increase of the communication range and with the decrease of the traffic flow density. 50% and 80% stops can be avoided if the communication range is over 400m and 700m, respectively. Most of the stops (90%) can be avoided under the V2V communication environment when the average space headway of vehicles is larger than 75m. Moreover, the rate of stops and total delay first increase and then decrease with the increase of the through movement percentage. The highest value occurs when the percentages of through and left turn are equal (50% to 50%).

The benefits of the V2V communication on the operational efficiency are closely related to the communication range, traffic flow density, and traffic flow pattern. The performance of the un-signalized intersection could be improved by lengthening the communication range. Moreover, more improvement can be obtained by the guidance strategy under lower traffic density and higher through movement percentage. Although all of results from this paper are based on simulation and sensitively analysis, in the further research, the real-world driving data should be collected to validate the proposed model.
